# Annotations

**Published:** 1892-01-23

**Authors:** 


### Annotations.

**Cardinal Manning.**

Cardinal Manning has passed away, leaving his country poorer by his death, but rich in the memory of his life. Although the man who has found the secret of science cannot for a moment hold the belief that any human being or aggregation of human beings, any Pope or any church, can be infallible; yet even men of science must recognise that Cardinal Manning was a man of the highest character, of great intellectual capacity, and of a profound benevolence which reached out to all mankind. Manning was bred in the Church of England, but at the mature age of forty-three went over to the Church of Rome. It is, perhaps, doubtful whether he would have taken that step if he had lived to see the extraordinary development of science and of historical criticism which has taken place during the last thirty years. He went over forty years ago, and in that forty years it may almost be said that a new faculty has been added to the human intellect—a faculty of patient enquiry, and of dispassionate and impartial acceptance of facts and their consequences. There is little doubt that Cardinal Manning, towards the close of his career, became less and less of a one-sided ecclesiastic, and more and more a Christian man of the world. There is no reason to doubt his loyalty to the church of his mature choice. But those who can read between the lines of his life think it was loyalty with a difference. He probably always regarded the Christian church as a divinely-established institution, as many of us do, and it is very likely he continued to think that on the whole the church associated with the Bishop of Rome had stronger claims to the adherence of minds that love both past and present than any other ecclesiastical organisation now in existence. But we do not for a moment believe that Manning regarded either the Pope or the Romish church as mechanically and absolutely infallible in the sense in which infallibility is ordinarily understood by the Protestant mind. It seems desirable to the scientific intelligence that this should be affirmed, because Manning was essentially a reasonable man, at any rate during his later years, and was willing to weigh the facts of science, and to give all reasonable consideration to the deductions which are properly to be drawn therefrom. All this is not said by way of attack upon the Catholic church, but by way of showing that capable minds may live honestly and righteously under what seem to other capable minds very peculiar restrictions. It is said also with the object of abating ecclesiastical acerbities by means of the sweetness and light of a true and patient science. The scientific man is sorry for the keen ecclesiastic. He wishes that every polemical divine had had the unspeakable advantage of a few years' scientific training in his mobile youth. Let us hope that science will ultimately be valued at its true worth as a means of liberal culture at our schools and universities. If it were, we should see the differences between cultured Jew, Gentile, Catholic, and Protestant gradually disappear. Science is the divine solvent which is destined to reduce all kinds of apparently diverse elements to a homogeneous and beautiful simplicity.

**A Famous Cancer Cure.**

There is at the present moment a general demand for specific cures for well-known fatal diseases, and the demand, as is common in the world's experience, creates a supply. The sale of so-called specifics for consumption, cancer, and other like remedies undoubtedly does a great deal of harm. It does not injure the medical profession so much as the public. People wonder sometimes why medical men inveigh against quack remedies and advertised specifics. But they need not wonder. Medical men know by personal experience how useless such things generally are; and they merely play the part of good neighbours when they warn the public against indulging hopes that are destined not to be realised, and also against wasting their money. The other day a friend sent to the writer a medical book which was published sixty-three years ago, and which gives an account of a cancer specific that has such an obvious bearing on the specifics of the present day that its reproduction in brief cannot but be of service to all really intelligent persons. Early in the present century Dr. Stoerck, of Vienna, published a medical work upon what he called the successful exhibition of hemlock in cases of confirmed cancer. Many of Dr. Stoerck's cures were vouched for by no less a person than the Baron Van Swieten, just as similar so-called cures are vouched for by well-known persons of position in the present day. Every medical practitioner was then, as now, eager to secure cancer cases upon which to experiment for himself with the new remedy. Dr. Akenside, of St. Thomas's Hospital, had been prescribing corrosive sublimate, with what he thought to be very gratifying success. But so desirous was he of securing still greater success, that he entirely gave up the corrosive sublimate treatment in order to work the wonders which the hemlock had been credited with in Vienna. He tried it on an extensive scale, and at every stage of the disease. At first, as is not unusual, his patients, buoyed up by a new hope, seemed to improve wonderfully, and his most sanguine expectations appeared about to be realised. Very soon, however, he found that the improvement ceased, and the disease in most of his cases began to progress as before. It is a striking fact that in tumours which had but just begun to develope, and where there was no ulceration, the marked improvement sometimes remained, and permanent cures were effected. That is to say, the new growth did not advance, but remained at a standstill, exactly as new growths often do now after many different kinds of treatment, and sometimes after no treatment at all. But in pronounced cases, where the cancerous ulcer had made considerable progress, the benefit of the treatment was very questionable. "It operated," says the chronicler, "often for a very few days like a charm, diminished the pains, and improved the discharge. But suddenly it failed any longer to do the slightest good, unless the dose were very largely increased. The dose was in many instances largely increased from time to time. At length the symptoms produced by the hemlock were as mischievous as those of the cancer itself, and Dr. Akenside was finally compelled to abandon its use altogether." This is a very instructive lesson for those who know how to profit by it. It coincides in almost every detail with the history of the so-called cures of the present day. The ignorant will no doubt long continue to be imposed upon by knaves, and those who may be rather fools than knaves. But the intelligent owe it to themselves to weigh evidence, and to preserve a sober and judicial mind.

**The Influenza Microbe.**

The epidemic of influenza, if it does nothing else that is of any service to man, promises to help the cause of bacilliary science, and through it, the progress of preventative medicine. Dr. Pfeiffer has discovered in the expectoration of influenza patients a peculiar small bacillus, which does not seem to be discoverable either in well persons or in those who are afflicted with any other disease than influenza. It is worth while to note the several important facts that Pfeiffer, Kitasato, and Canon, working under the general supervision of Koch, have brought to light. The small bacillus which those laboratory workers suppose to be the cause of influenza is, as we have said, found in influenza patients, but not in other persons. It is present in the mucus of the nose and throat, and its numbers vary with the intensity of the catarrhal process. It is present, moreover, in the blood of persons suffering from influenza, and here, also, its numbers vary with the severity of the symptoms of the disease. Not only so, but in certain cases where pleurisy has supervened upon the influenza, the microbe is found in the fluid exuded into the chest cavity; and here, as before, the numbers of the microbes are directly proportionate to the severity of the pleuritis and to the quantity of exuded fluid. All the natural fluids and semi-fluids of the body, and the fluids and the semi-fluids that are the products of disease seem to be equally infested with the influenza microbe in cases of true influenza, and to be so infested in the case of no other known disease. It is evident, therefore, that whether this particular microbe be or be not the actual cause of the disease, it is at least a constant accompaniment of it. There is, in fact, a reasonable presumption that it is the cause. Moreover, the injection of this bacillus into certain animals is said to have produced true influenza. If the microbe, and the microbe only, be proved to be the *vera causa* of influenza, then we have the prevention of the disease in an epidemic form largely in our own hands. Isolation, immediate and complete, in every case is our only safety. No doubt much more remains to be done before we can feel assured that Pfeiffer and his fellow-workers are on the right tack; but if it should ultimately be proved that they are, influenza, like other infectious diseases, will be no longer an unknown and all-conquering foe, but an enemy subdued and, to a large extent, safe under lock and key.

